# Possible etiology of improvements in both quality of life and overlapping gastroesophageal reflux disease by proton pump inhibitor treatment in a prospective randomized controlled trial

**DOI:** 10.1186/1471-230X-13-145

**Published:** 2013-10-01

**Authors:** Hubert Mönnikes, Thomas Schwan, Christo van Rensburg, Andrzej Straszak, Carmen Theek, Reinhold Lühmann, Peter Sander, Anne Tholen

**Affiliations:** 1 Department of Medicine and Institute of Neurogastroenterology, Academic Teaching Hospital Martin Luther, Charité - Universitätsmedizin Berlin, Caspar-Theyß-Str. 27-31, Berlin, 14193, Germany; 2 Nycomed GmbH, Nycomed: a Takeda Company, Konstanz, Germany; 3 Tygerberg Academic Hospital, Stellenbosch University, Cape Town, South Africa; 4 Department of Internal Medicine, City Hospital, Siemianowice Śląskie, Poland; 5 Pierrel Research, Essen, Germany; 6 Takeda Pharmaceuticals International GmbH, Zürich, Switzerland

**Keywords:** Gastroesophageal reflux disease (GERD), Functional dyspepsia (FD), Irritable bowel syndrome (IBS), Inflammatory bowel diseases (IBD), Proton pump inhibitor (PPI)

## Abstract

**Background:**

Symptoms suggestive of functional dyspepsia (FD) and irritable bowel syndrome (IBS) frequently overlap with those of gastroesophageal reflux disease. Despite the high prevalence of symptomatic overlap, the underlying etiology remains poorly defined. We assessed the correlation of symptomatic relief and health-related quality of life (HRQoL) with healing of reflux esophagitis to further derive insights into the underlying etiology.

**Methods:**

626 patients with reflux esophagitis were enrolled into one of two treatment groups (classical healing concept or the complete remission concept) to investigate differences in treatment intensity. Patients were treated with pantoprazole until esophageal mucosal healing. Remission was followed for up to 6 months without treatment. Gastro-intestinal symptoms and HRQoL were analyzed using disease-specific, psychometrically validated patient-reported outcome instruments (ReQuest™, GERDyzer™).

**Results:**

Symptomatic burden reflected by ReQuest™ substantially decreased from baseline to end of treatment by 83% and 88% in either treatment group, respectively. ReQuest™ scores significantly decreased in patients with or without heartburn and in those with symptoms suggestive of FD and IBS, indicating response of all symptom categories to treatment (p < 0.005). Therapy-associated relief of symptoms was paralleled by substantial gains in HRQoL, which continued to stabilize post-treatment.

**Conclusions:**

Pantoprazole is effective in relieving upper and lower gastro-intestinal symptoms overlapping with erosive esophagitis, and provides sustained improvement in HRQoL post-treatment. Our results propose a link between both healing of erosive esophagitis and the slower remission of upper and lower gastro-intestinal symptoms. Since the improvement observed is likely to be multifactorial, the possibility for an immune-mediated etiology and identification of putative susceptibility factors by genome-wide association study may provide focus for future research.

**Trial registration:**

ClinicalTrials.gov identifier: NCT00325676.

## Background

Gastroesophageal reflux disease (GERD) is highly prevalent in the general population. In most cases, GERD is considered a chronic disorder requiring long-term acid suppression to maintain symptomatic remission and/or healing of erosive esophagitis [1,2]. GERD significantly impacts on health-related quality of life (HRQoL) [3,4]. Veldhyzen van Zanten et al. (2012) reported a close association between increased patient satisfaction, relief of GERD symptoms, and improved HRQoL, with relevant associations similar to those in other chronic diseases [5]. Additional investigations have been conducted for functional bowel disorders such as irritable bowel syndrome (IBS), concluding that therapeutic response is associated with improvements in HRQoL [6]. These reports focus on qualitative or statistical correlations, but do not propose any mechanistic psychological concept for the observed concordance.

Symptomatic overlap of GERD with gastro-intestinal (GI) co-morbidity suggestive of functional dyspepsia (FD) or irritable bowel syndrome (IBS) [7-10] is thought, amongst other explanations, to contribute to partial or complete non-response to a standard dose of proton pump inhibitor (PPI) in a significant subset of patients [3,5,11]. However, overlap does not appear to be static over time as symptom categories tend to fluctuate during long-term follow-up [8,12]. Causative patho-mechanisms currently considered include inflammatory etiologies [13,14], altered intestinal motility [15], and visceral hypersensitivity [16], each suggesting the need for individualized therapeutic approaches in specific patient groups.

Cumulative clinical and scientific discoveries, along with regulatory requirements of demonstrating statistical significance for efficacy claims, have resulted in increasingly narrowly defined indications and stratified patient populations in clinical trials. Since such an approach might not reflect the actual patient population seeking medical help, it might be useful to study the pharmacological management of a real-life scenario [17], which would allow for the investigation of an overlap between GERD and symptoms suggestive of FD and IBS. Although such a study might carry the elevated risk of observing only a partial response to PPI treatment in these less stringently selected patients [5], such a design could reveal additional insights into the inter-relationship between perceived symptoms and HRQoL, and the pathophysiological relatedness of, or separation between, GERD, FD, IBS and possibly early-stage inflammatory bowel diseases (IBD).

In the current study, we therefore investigated a patient population that closely resembles the general population affected by endosopically-confirmed GERD, which overlaps with upper and lower GI symptomatology. In addition, we evaluated two treatment groups, which modelled differences in treatment intensity and compliance. We further hypothesized that treatment intensity would influence patterns of relapsing erosive esophagitis, and that the analysis of the temporal development of overlap during treatment and remission could shed additional insights into an underlying etiology.

## Methods

### Study design and patients

**Figure 1:**
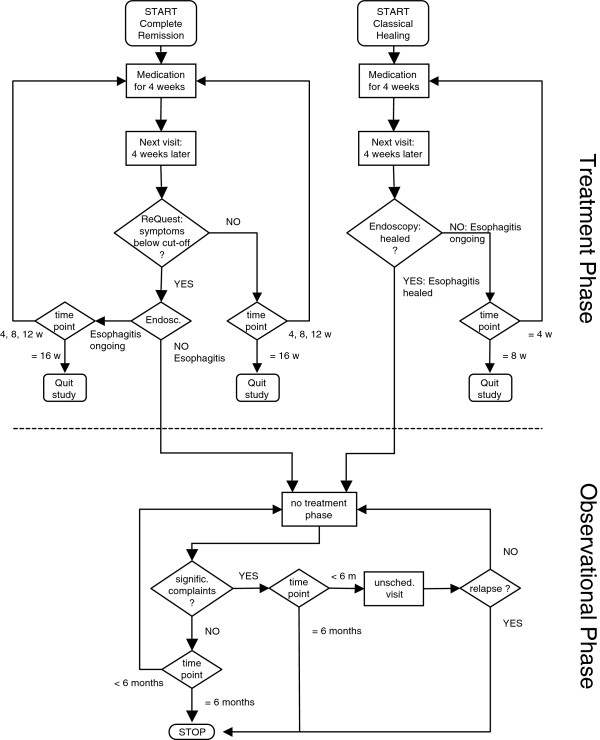
**Treatment algorithm.** START, V0; STOP, last visit of Observational Phase; m, months; w, weeks.

The design and primary results of this prospective clinical trial (ClinicalTrials.gov identifier: NCT00325676) have been previously described elsewhere [18]. This report includes prospectively defined subgroup analyses of therapy-associated changes of GI symptomatology and HRQoL. In brief, this randomized, multicenter, multinational, parallel group study (June 2006 to May 2007) was conducted in patients with GERD diagnosed endoscopically according to the Los Angeles classification (LA grade A–D) (full inclusion and exclusion criteria have been previously presented [18]). To assess the treatment effects based on duration and intensity of PPI administration, patients were randomized to two different treatment concepts: classical healing (CH) and complete remission (CR) (Figure 1). The CH group was treated up to 8 weeks until esophageal healing only (according to the LA classification), whereas the CR group was treated up to 16 weeks until 'complete remission’ occurred. This was defined as esophageal healing and a ReQuest™ (Reflux Questionnaire, Nycomed GmbH, Nycomed: a Takeda Company, Konstanz, Germany) -GI score below the predefined symptom threshold [19]. The trial was further divided into two periods: a treatment phase and a subsequent observational phase. During the treatment phase, patients were administered pantoprazole 40 mg od until resolution of GERD as pre-specified for each group. Healed patients entered an observational phase, which consisted of clinical follow-up for up to 6 months without PPI treatment and monitoring for endoscopically-confirmed relapse of reflux esophagitis according to the LA classification. To further assess concurrent symptoms affecting the upper and lower GI tract, a questionnaire was implemented asking for heartburn and modified symptomatic Rome II/III criteria, the latter suggesting diagnoses of FD and IBS [18].

The trial was performed in accordance with the Declaration of Helsinki and the International Conference on Harmonisation-Good Clinical Practice (ICH-GCP), and was approved by the independent ethics committees (Commission of Bioethics of Silesian Medical Chamber in Katowice [Poland]; Pharma Ethics, and University of the Free State Ethics Committee [South Africa]; Ärztekammer der Ethikkommission Schleswig-Holstein [Germany]) for each participating center. Prior to participation, all patients provided written informed consent.

### Assessments of GI symptoms (ReQuest™)

The course of GI symptom profiles was measured over time using ReQuest™. ReQuest™ is a disease-specific, self-administered and psychometrically validated patient-reported outcome instrument designed to assess the course of GERD symptoms [19]. It is divided into two subscales, ReQuest™-GI (including dimensions 'acid complaints’, 'upper abdominal/stomach complaints’, 'lower abdominal/digestive complaints’ and 'nausea’) and ReQuest™-WSO (assessing dimensions 'general well-being’, 'sleep disturbances’ and 'other complaints’). Dimensions were assessed for frequency and intensity, except for 'general well-being’ where only intensity was recorded.

### Assessments of HRQoL (GERDyzer™)

Treatment-induced changes in HRQoL were assessed using GERDyzer™ (GERD Analyzer Scale, Nycomed GmbH, Nycomed: a Takeda Company, Konstanz, Germany). GERDyzer™ is a disease-specific, self-administered and psychometrically validated patient-reported outcome instrument that was developed to investigate the impact of GERD on HRQoL [4]. GERDyzer™ comprises 10 dimensions of quality of life: 'general well-being’, 'pain/discomfort’, 'physical health’, 'energy’, 'daily activities’, 'leisure activities’, 'social life’, 'diet/eating/drinking habits’, 'mood and sleep’, and satisfaction aspects.

### Statistical analysis

In general, descriptive summary statistics were determined showing parameters for location and dispersion for continuous parameters, and frequencies and percentages for categories. The course over time for mean values of parameters is displayed graphically by line plots comparing the respective groups of patients.

ReQuest™ total scores were analyzed using descriptive statistics. Between-group comparisons for patients with or without heartburn, with or without FD, and with or without IBS were performed using the Wilcoxon rank-sum test.

HRQoL measured by GERDyzer™ was evaluated using explorative methods. The main analysis investigated GERDyzer™ mean sum scores. Sum scores pertaining to the observational phase were compared between both treatment groups using the Wilcoxon rank-sum test.

## Results

### Patient characteristics

Of the 634 patients who initially received study medication, 316 (49.8%) were treated according to the CH concept and 318 (50.2%) according to the CR concept. Out of the safety set of 634 patients, 539 individuals (85.0%) were diagnosed with erosive esophagitis LA grade A–B and 95 individuals (15.0%) with erosive esophagitis LA grade C–D. The intention-to-treat (ITT) population comprised 626 patients, with 313 patients being allocated to each of the two treatment concepts (CH or CR). Of these, 87 patients were enrolled in Poland (CH 45, CR 42), 301 in South Africa (CH 160, CR 141), and 238 in Germany (CH 108, CR 130). Most patients were of white ethnicity (CH 78.6%, CR 80.8%). FD-like complaints were diagnosed at baseline in 65.2% and 64.2%, and IBS-like co-morbidity existed in 14.1% and 13.1% of the CH and CR groups, respectively (Table 1). The number of patients in subgroups of FD and subgroups of IBS tended to be rather small (data on file). Approximately one-quarter of patients in each treatment group were current smokers. Neither ReQuest™ total scores nor GERDyzer™ sum scores were notably different at baseline in the treatment arms.

**Table 1:** Demographic data and clinical disposition at baseline (ITT, N = 626)

**Variable**		**CH group**	**CR group**
		**(N = 313)**	**(N = 313)**
Gender, *n* (%)	Male	167 (53.4)	170 (54.3)
	Female	146 (46.6)	143 (45.7)
Age [years], mean (SD)		50.8 (14.6)	48.8 (14.0)
Weight [kg], mean (SD)		81.8 (16.4)	83.4 (17.4)
BMI [kg/m^2^], mean (SD)		28.0 (5.1)	28.6 (5.7)
Race, *n* (%)	Asian	14 (4.5)	7 (2.2)
	Black	13 (4.2)	17 (5.4)
	White	246 (78.6)	253 (80.8)
	Other	40 (12.8)	36 (11.5)
GI comorbidities, *n* (%)	Dyspepsia-like	204 (65.2)	201 (64.2)
	IBS-like	44 (14.1)	41 (13.1)
Current smoker, *n* (%)		79 (25.2)	83 (26.5)
ReQuest^TM^ total score		10.23	11.37
GERDyzer^TM^ sum score		46.12	49.45

*CH* classical healing, *CR* complete remission, *IBS* irritable bowel syndrome, *ITT* intention-to-treat, *N* number of patients, *BMI* body mass index, *SD* standard deviation.

### Therapy-associated change in GI symptoms

**Figure 2:**
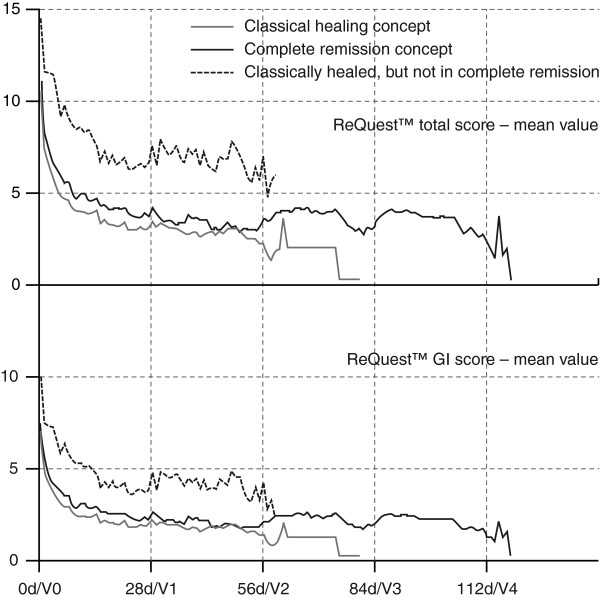
**Change in gastrointestinal (GI) symptom burden.** ReQuest^TM^ total score & GI score; d, days.

The change of the GI symptom burden over time as evaluated by ReQuest™ is shown in Figure 2. For both treatment groups, the ReQuest™ total scores were significantly lower at the end of pantoprazole treatment (CH 2.14, CR 1.76) than at baseline (CH 10.23, CR 11.37). This resembles a clinically significant decrease of the total score by 79% in the CH group and by 85% in the CR group. This finding was paralleled by a similar time course for the two subscales ReQuest™-GI and ReQuest™-WSO. The ReQuest™-GI score substantially decreased from baseline (CH 6.74, CR 7.29) to the end of treatment (CH 1.12, CR 0.84), reflective of a decrease of the GI score by 83% in the CH group and by 88% in the CR group (Figure 2).

To evaluate the influence of concurrent symptoms affecting the upper and lower GI tract, a joint analysis of both treatment groups was performed. At baseline, higher ReQuest™ total scores were identified in patients with concurrent symptoms suggestive of FD (12.45) or IBS (14.47) than in patients with (11.37) or without (5.53) heartburn. At the end of pantoprazole treatment, ReQuest™ scores notably decreased in patients with (4.65) or without heartburn (1.41), and in those with symptoms suggestive of FD (4.36) or IBS (4.04), indicating response of all symptom categories to PPI treatment. Interestingly, the trend for higher total scores at baseline from concomitant heartburn (11.37) to IBS (14.47) was inverted post-treatment (heartburn 4.65, IBS 4.04). Comparison of ReQuest™ total scores among groups of patients with or without heartburn, FD, or IBS was statistically significant (all, p < 0.005).

In order to further evaluate the appropriateness of both healing concepts, courses over time of the ReQuest™ total score and its subscales were plotted for both the CH and CR groups in conjunction with total scores of a subgroup deduced from CH patients who were endoscopically healed but not in complete remission. As can be seen from Figure 2, ReQuest™ scores were almost 2-fold higher for patients endoscopically healed but not in complete remission than for patients in either the CH or CR groups, suggesting the clinical relevance of treatment aimed at full symptomatic remission in addition to esophageal mucosal healing.

### Therapy-associated change in HRQoL

**Figure 3:**
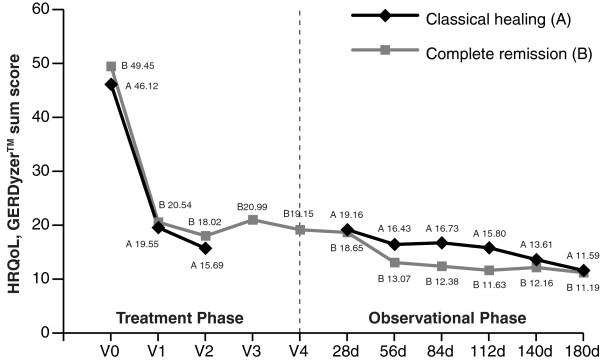
**Change in health-related quality of life.** HRQoL, GERDyzer^TM^ sum score during the treatment phase and the observation phase of the study; d, days **(A)**, classical healing group; **(B)**, complete remission group.

Data from the HRQoL questionnaire GERDyzer™ were available from 606 patients at baseline, with data from 3.3% of patients missing (ITT). Overall, the significant decrease in GERDyzer™ mean sum scores during pantoprazole treatment indicated that HRQoL improved in both the CH and CR study groups (Figure 3). At the end of the treatment phase, GERDyzer™ mean sum scores decreased by about two-thirds in each of the two treatment schedules investigated, with most of the effect achieved during the first 4 weeks of pantoprazole treatment (i.e., at the first study visit post-baseline). Substantial gains in HRQoL resulted from a major decrease in all 10 specific dimensions of GERDyzer™ (data on file). There was no significant difference in GERDyzer™ sum scores between the two groups studied. HRQoL measured by GERDyzer™ further improved for the CH and CR group during the observational phase (Figure 3), as reflected by decreasing mean sum scores. Although patients treated according to the CR concept consistently had lower GERDyzer™ sum scores, suggesting continuously improved HRQoL, exploratively evaluated p-values were greater than the α-level of 0.05, indicating no significant difference in HRQoL between the two treatment concepts studied.

In order to further assess both healing concepts regarding improvements in HRQoL, GERDyzer™ scores for the CH and CR groups were analyzed in relation to scores of a subgroup deduced from CH patients who were endoscopically healed but not in complete remission (i.e., not achieving symptomatic relief to a level of healthy individuals). As with the ReQuest™ scores for these three groups, GERDyzer™ scores in the CH and CR groups were approximately only half the scores of patients endoscopically healed but not in complete remission, thus indicating notably impaired HRQoL in patients achieving esophageal healing only (data on file).

### Adverse events

Because patients randomized to CR remained in the active treatment phase notably longer than patients allocated to CH, a direct comparison of the incidence of adverse events has to be conducted carefully. Duration of exposure in the CH group was 42.3 ± 14.8 days compared with 56.8 ± 31.5 days in the CR group (ITT), which resembles an increase in exposure from CH to CR of 34%. Of the 634 patients in the safety set, 175 (27.6%) reported at least one treatment-emergent adverse event (TEAE), most of which were mild-to-moderate in intensity. A total of 81 of 316 patients (25.6%) in the CH group and 94 of 318 patients (29.6%) in the CR group experienced at least one TEAE, reflective of a difference in TEAE of 4% between the two groups studied. In relation to the increase in exposure of 34%, a parallel increase in TEAEs of 4% suggests that most TEAEs were not causally related to the intake of pantoprazole. This was further substantiated by the investigators’ causality assessment, in which 84% of TEAEs in the CH group and 79% of TEAEs in the CR group were considered as 'not related’ or 'unlikely related’ to the intake of the study medication.

## Discussion

Clinical trials often include narrowly defined clinical indications and thus patient populations in order to meet regulatory or scientific requirements. In contrast, in the present study we used a scenario that was closer to real-life to investigate clinical and psychometric responses to PPI treatment in patients with endosopically-confirmed GERD [17]. This was achieved by: (1) evaluating two different treatment concepts that modelled differences in treatment intensity and compliance; and (2) applying broad inclusion criteria, which allowed assessment of overlapping GI conditions that frequently occur in selected patients and in the general population [8-10,20]. Differential assessment of patient-reported outcomes by ReQuest™ indicated that endoscopically healed patients who did not achieve symptomatic remission to the level of healthy individuals continued to suffer from a substantial GI symptom burden. These individuals had symptom scores that were 2-fold higher than those in patients with endoscopic healing and symptomatic remission. This was associated with notably reduced quality of life, as indicated by scores of GERDyzer™ that were approximately 2-fold higher than those in patients with both endoscopic healing and symptomatic remission. However, this differential treatment response was not evident in the direct comparison of the original treatment groups (CR and CH), for which there was no significant difference [18]. This might be a reflection of the number of patients who at each 4-week assessment point were already in symptomatic remission and were screened for endoscopic healing only. Nevertheless, the potential clinical significance of the CR concept was revealed by the cautiously implemented, pre-specified, secondary assessment of a differential subgroup of CH patients with endoscopic healing who did not fulfil the CR criteria.

Taken together, the analyses suggest: (1) a close correlation between HRQoL and perceived GI symptoms in GERD patients, which is consistent with previously published research [6,20]; and (2) the clinical relevance of a sufficient treatment effort aimed at symptomatic remission in addition to healing of the esophageal mucosa, i.e., until complete remission is achieved. The latter finding is further substantiated by a recently reported long-term clinical trial investigating pantoprazole in tertiary care patients over the course of 15 years [2]. Analysis indicated that the mean duration until relapse can be prolonged to approximately 7 years by the continuous administration of acid-suppressing medication.

To the best of our knowledge, we report for the first time that PPI therapy can provide improvements in HRQoL which continued to be maintained post-treatment. This occurred despite the chronic nature of erosive esophagitis in most patients [1]. However, this observation might be a consequence of the close correlation between perceived symptoms and HRQoL, and the clinically meaningful difference developing towards the end of the observational phase between the rates for relapsing erosive esophagitis (65%) and GERD symptoms (36%), indicative of a symptomatically silent disease in many patients despite visible damage of esophageal mucosa [18,21]. Thus, the delayed increase in GERD symptoms following relapse of erosive esophagitis appears to be mirrored by a trend for continuously improved HRQoL during the observational phase, until a point in time after endoscopic relapse is met when GERD symptom-related afferent input into the central nervous system exceeds the threshold for entering awareness. This would then trigger a coherent cognitive representation of chronically persisting or remitting symptoms, and thus provide the emotional basis for reduced HRQoL.

The high number of patients in our study with baseline dyspeptic (65%) and/or IBS-like complaints (14%) that resemble the types of symptoms generally attributed to functional GI disorders (FGID), such as FD and IBS, is of note. Inter-current dyspeptic and IBS-like complaints, overlapping with reflux esophagitis and GERD symptoms has been previously described in selected populations and in the general population [8-10,20]. Our data, along with those of others [20], indicate significantly increasing ReQuest™ scores (reflecting increasing symptomatic burden) of GERD overlapping with dyspeptic and/or IBS-like complaints. After treatment with pantoprazole, rates of patients fulfilling symptomatic Rome criteria [18] and ReQuest™ scores were generally substantially lower, indicating response of all symptom categories to PPI treatment.

Given the overlap of GERD with dyspeptic and/or IBS-like complaints, the question of whether or not individual FGID are separate conditions from GERD has been raised, and consequently different hypotheses for a possible underlying etiology have been proposed [13-16,18]. Etiological hypotheses for FGID include low-level or transient inflammation of part of the GI tract [13,14], altered intestinal motility [15] or visceral hypersensitivity [16], each of which suggests the need for different therapeutic approaches in specific patient groups. Ford et al. (2010) describe a thoroughly investigated case of a more than 2-fold increase in the prevalence of dyspepsia in patients suffering from transient gastroenteritis 8 years prior to conducting their investigation [13]. One conclusion provided by the authors is that inflammation in one part of the GI tract could induce changes of the enteric nervous system of a non-inflamed part, a finding previously observed in animal models. Similar reports in humans are available on a putative pathogenic role of low-grade inflammation, identifying increased counts of mast cells in both the colon and upper GI tract of subjects with IBS, potentially leading to an abnormal interaction between mast cells and nerve fibers [14]. Such findings showing interactions between distant parts of the GI tract via the enteric nervous system or cells of the immune system may provide an explanation as to why FD and IBS were described by others as longitudinally unstable, fluctuating disease entities [8,12]. This cellular cross-talk might at least partially explain why improvements in reflux esophagitis have beneficial effects on overlapping symptoms suggestive of FD and IBS [18].

Evidence from distant disciplines such as molecular biology, immunology and clinical research converges on abnormal interactions between the host and the external human microbiome in the pathogenesis of IBS [14] and IBD [22], establishing an etiological interface between IBS and IBD [23]. Molecular interaction between host cells and commensal enteric bacteria, or transient acute gastroenteritis induced by specific pathogens, could lead to a continuous activation of cells of the innate and acquired immune system causing chronically perceived symptoms or lasting injury to part of the GI tract. The efficacy of antibiotic therapy in IBS and IBD provides some support for such a patho-mechanism [22,23], although theoretical interest in small intestinal bacterial overgrowth is decreasing [23]. However, our observation that FD and IBS symptomatology improve significantly during PPI treatment, although at a slower rate than the signs and symptoms of GERD [18], could possibly favour an underlying mechanism that is primarily immune-mediated rather than based on neural process. Such an immune-mediated patho-mechanism would depend on sufficient time for the clonal selection, migration, homing and peripheral dispersion of immune cells. Further support for an immune-mediated patho-mechanism is provided by studies that have investigated cellular and cytokine profiles in GERD, along with the effect of PPI treatment on them [24,25]. In such studies, PPI therapy suppressed the expression of pro-inflammatory cytokines and chemokines, such as interleukin-8 (IL-8). Complementary studies demonstrated a reduction of the cellular-mediated inflammatory infiltrate in the esophageal epithelium, as represented by significantly decreased numbers of T lymphocytes and CD8+ T lymphocytes during PPI therapy. Interestingly, both the levels of IL-8 and the number of T lymphocytes have been shown to correlate with endoscopic severity of reflux esophagitis [24,25].

Another etiological hypothesis meriting discussion is the theoretical potential for psychiatric co-morbidity, serving as a default diagnosis in patients complaining about idiopathic, unpleasant perceptions or pain located in the GI tract [26]. Such psychological disorders are described as often preceding the onset of IBS, suggesting they should not be considered as a consequence of suffering primarily from IBS [26,27]. However, GI complaints subsequent to confirmed psychiatric morbidity could be adverse drug reactions resulting from the use of psychotropic medication; they would thus be nosologically different to suffering from a functional GI disorder. Known effects of psychotropic medication occur at a compound-specific level and are variable across individual patients [28]: (1) modulation of sensory processing, involving e.g., taste [29,30], vision [31,32] and pain [33]; (2) manipulation of the endogenous emotional homeostasis [28,34-36]; and (3) the generation of further adverse reactions reported to significantly affect the GI tract, including bleeding [37], constipation [38-43] and diarrhea [39,40,42]. Unfortunately, it is not always obvious from published reports whether or not studies have been subject to potential confounding by the concomitant use of psychotropic medication [27], or if anxiety and depression might provide part of the natural spectrum of an idiopathic, chronic disease being accompanied by pain or marked discomfort [20].

FGID and GERD are reported to cluster in families [14,44-46]. Consequently, if an etiological genetic contribution could be proven by identifying polymorphisms of causative genes, baseline demographic data of contemporary clinical trials, such as that provided in Table 1, are currently missing the evaluation of the genetic composition of enrolled patients. However, the challenges of both determining a relevant set of genes and those of a meaningful, medically actionable, integrative analysis are well known [44,45,47,48]. In addition, different interpretations of competing or additive contributions of both a genetically imprinted trait matrix [48] and environmental health modifiers are amply discussed in the literature. Nevertheless, the identification of key genes could be attempted by conducting a sufficiently powered genome-wide association study on FGID, investigating reasonably selected patients and conditions [48]. Omitting the known conceptual limitations of candidate gene approaches, the virtue of such an investigation would be the identification of disease predisposing loci not being limited to, or excluded by, specific *a priori* etiological hypotheses.

Our study provides both several strengths and certain limitations. Our study enrolled a large patient sample, avoiding narrowly defined inclusion and exclusion criteria, thus being close to a real world medical setting. This offered the opportunity of studying interrelations between both endoscopically-confirmed GERD and symptoms suggestive of FGID, and more psychometrically definable conditions like HRQoL. However, due to the focus on endoscopically-confirmed GERD (LA grades A–D), patients with endoscopy-negative GERD or those with purely functional symptoms were not included in our investigation. Despite the elaborate patient sample studied, specific subtypes of FD and IBS still tended to be relatively rare.

## Conclusions

Therapy-associated changes of GI symptomatology broadly correlated with HRQoL in patients with an overlap of endoscopically-confirmed GERD and symptoms resembling FD and IBS. Furthermore, HRQoL appears to be negatively correlated with endoscopic relapses of erosive esophagitis, with the latter generally presenting initially as a silent condition. From an etiological perspective, interactions between distant parts of the GI tract via the enteric nervous system or cells of the immune system may provide an explanation for independent reports of FD and IBS presenting as longitudinally unstable, fluctuating conditions. Since the improvement observed is likely to be multifactorial, this mechanistic concept may partly explain the parallel improvements in reflux esophagitis and overlapping symptoms suggestive of FD and IBS. The previously reported observation of slower improvements in symptoms suggestive of FD and IBS could hint to a primarily immune-mediated, rather than neural, etiology, which potentially relies on the known time-requiring steps involved in clonal selection, migration, homing and peripheral dispersion of immune cells. Nevertheless, hypothesis-free exploration of underlying patho-mechanism(s) and investigating a potential molecular interface towards IBD should justify conducting a sufficiently powered and well-designed genome-wide association study. This could be conducted both in patients suffering from functional GI disorder and in those suffering from GERD with and without symptomatic overlap.

## Competing interests

HM has served as speaker, consultant, and advisory board member for ALTANA, Astra Zeneca, Falk, Novartis, Nycomed GmbH (formerly ALTANA Pharma), Solvay, Steigerwald, and Wyeth. He received research funding from Nycomed GmbH (formerly ALTANA Pharma), Charité, DFG, Medtronic, and Sonnenfeld Foundation. HM owns no stocks and shares in pharmaceutical companies. CvR has no personal or funding interests. CT works for Pierrel Research. AS has served as speaker and/or consultant for ALTANA, Falk, Ferring, Janssen-Cilag, MSD, Nycomed, and Solvay. TS, RL, PS, and AT are employees of Nycomed GmbH (Nycomed: a Takeda Company).

## Authors’ contributions

HM, TS, PS and RL designed the clinical trial and wrote the manuscript draft. HM, TS, PS, RL and AT were involved in data analysis and preparation of the manuscript. CT contributed to the planning and evaluation of the clinical trial. CvR and AS were involved in the clinical conduct and critically revised the manuscript. All authors read and approved the final manuscript.

## Pre-publication history

The pre-publication history for this paper can be accessed here:

http://www.biomedcentral.com/1471-230X/13/145/prepub
